# An Improved YOLOv9 Based Object Detection with Attention Mechanism for Personal Protective Equipment

**DOI:** 10.3390/s26103058

**Published:** 2026-05-12

**Authors:** Geunho Lee, Jieun Lee, Tae-yong Kim, Jongpil Jeong

**Affiliations:** Department of Smart Factory Convergence, Sungkyunkwan University, Suwon 16419, Republic of Korea; ohoylee@gmail.com (G.L.); lu3873@g.skku.edu (J.L.); skywin94@naver.com (T.-y.K.)

**Keywords:** attention mechanisms, industrial safety applications, object detection, personal protective equipment, YOLOv9

## Abstract

Industrial sites pose numerous hazards where unexpected accidents can occur at any time, and personal protective equipment (PPE) is a primary safeguard for worker safety. In this study, PPE specifically refers to safety helmets, safety shoes, and safety gloves. Manual verification of PPE usage is infeasible in environments with many workers, motivating automated detection. This study proposes a method that integrates the Convolutional Block Attention Module (CBAM) exclusively into the training-only auxiliary reversible branch of YOLOv9’s Programmable Gradient Information (PGI) architecture. The proposed CBAMLinear module enhances gradient information during training while introducing zero additional computational overhead at inference, as the entire auxiliary branch is removed. The proposed YOLOv9 with CBAMLinear achieved consistent mAP@0.5:0.95 gains of 0.005–0.007 over the baseline for the three larger model variants, while maintaining identical inference-time parameters and FLOPs. In industrial safety, even modest performance gains can directly contribute to accident prevention by reducing false positives and false negatives, making this approach well suited for real-time safety management systems in industrial settings.

## 1. Introduction

Industrial workplace accidents remain a significant concern in the Republic of Korea. According to recent epidemiological analysis, the construction sector alone accounts for 51.9% of all industrial fatalities, with a mortality rate of 2.48 per 10,000 workers—more than twice the national industrial average [1]. The Korea Occupational Safety and Health Agency further reports that construction sites generate 46.7% of all occupational fatalities and 25.5% of all occupational injuries nationwide [2]. In industries such as manufacturing and construction, the wearing of personal protective equipment (PPE) is a critical matter directly linked to workers’ lives, as failure to wear PPE can lead to personal injury, property damage, and secondary accidents. However, most industrial sites still rely on limited human resources or closed-circuit television (CCTV) monitoring to verify PPE usage, an approach that becomes inadequate as site scale and worker count increase. Field blind spots and continuous human monitoring limitations inevitably introduce errors, motivating the need for automated detection systems [3].

With recent rapid technological advances in artificial intelligence and computer vision, object detection technology has gained considerable attention as a possible solution to this problem in the industrial field. In particular, the YOLO (You Only Look Once) model has emerged as a fast and accurate way to detect objects by processing the entire image at once [4]. Unlike existing complex detection methods, YOLO predicts the location and type of multiple objects in an image simultaneously through a single neural network structure. This enables YOLO to achieve satisfactory performance with both high speed and high accuracy [5]. However, in real-world industrial settings, challenges such as ever-changing lighting conditions, complex backgrounds, varying worker postures, and frequent occlusions arise. As a result, standard YOLO models produce false positives and false negatives, undermining the reliability of safety management systems.

First, existing approaches that integrate attention mechanisms into the YOLO architecture introduce additional computational overhead during inference. Methods such as YOLOv8-CGS, which combines CBAM and Global Attention Mechanism (GAM) with the main detection architecture, and MEAG-YOLO, which integrates multi-scale channel attention into the feature extraction network, have improved accuracy but at the cost of increased inference latency and model parameters [6,7]. The trade-off between accuracy and real-time performance acts as a significant constraint in the practical implementation of industrial safety monitoring systems, where low-latency processing is essential. Second, detecting PPE in real-world industrial settings remains a challenging task due to the small size of the target objects, complex and cluttered backgrounds, varying lighting conditions, and frequent occlusion. Items such as safety gloves or safety shoes occupy an extremely small portion of the image, making them difficult to distinguish from the surrounding background. Third, there is currently no method to solve these two challenges simultaneously. Existing attention-based approaches achieve high detection accuracy but suffer from slow inference speeds. Conversely, lightweight models maintain speed but lack the ability to reliably detect small PPE objects in complex scenes. Therefore, an approach is needed that can improve training quality without compromising the real-time inference performance of the base detection model.

This study proposes a method combining YOLOv9 and the Convolutional Block Attention Module (CBAM) to improve the detection accuracy of personal protective equipment in industrial settings. While YOLOv9 minimizes information loss and enhances computational efficiency through Programmable Gradient Information (PGI) and Generalized Efficient Layer Aggregation Network (GELAN), additional measures are required to mitigate environmental factors effectively, such as complex backgrounds, varying lighting conditions, and object occlusion in industrial settings [8].

YOLOv9 was selected as the base architecture due to its unique PGI (Programmable Gradient Information) structure, which includes auxiliary reversible branches that are activated only during training and completely removed via reparameterization during inference. Thanks to this architectural feature, an attention module can be inserted without incurring additional computational costs during inference. In contrast, while YOLOv10 adopts a dual-head structure for inference without NMS (Non-Maximum Suppression), this functions purely as a label assignment strategy rather than a parallel feature extraction path [9]. Inserting an attention module into YOLOv10 directly increases inference overhead in either the backbone or the neck. Similarly, since YOLO11 already integrates C2PSA (Cross-Stage Partial with Parallel Spatial Attention) into its architecture, introducing an additional attention mechanism would inevitably increase inference costs [10]. Among recent YOLO variants, YOLOv9’s PGI architecture is structurally distinguished by maintaining a training-only auxiliary reversible branch that is removed at deployment via reparameterization. This dual-branch design enables hosting attention modules in a location that does not affect the deployed inference graph—a property that, in the standard configurations of single-branch detectors such as YOLOv10 and YOLOv11, is not directly available without architectural modification. This structural property is empirically verified in Section 4, where the Parameter, FLOPs, and FPS of every YOLOv9 variant are shown to be identical with and without CBAMLinear, confirming zero deployment overhead by direct measurement.

To achieve this, this study integrated CBAM into the CBLinear module within the PGI auxiliary reversible branch of YOLOv9, creating CBAMLinear. CBAMLinear learns sequentially to emphasize important features in the channel and spatial dimensions during training to clearly distinguish personal protective equipment and is deactivated during inference, incurring no computational cost increase. Experimental results, including the parameter and FLOPs measurements presented in Section 4, indicate that the proposed method improves detection performance compared with existing YOLO models while maintaining identical inference-time computational cost to the baseline YOLOv9, demonstrating its suitability for integration into real-time safety management systems in industrial settings.

The main contributions of this study are summarized as follows. First, we propose a resource-efficient inference paradigm that inserts the CBAM module only into the auxiliary reversible branch within the PGI architecture of YOLOv9. Unlike existing approaches that embed attention mechanisms into the main inference architecture, the proposed CBAMLinear module enhances gradient quality and feature representations during training while remaining completely inactive during inference, thereby maintaining the same computational cost as the baseline model, YOLOv9. Second, we present an architectural analysis showing that YOLOv9’s PGI structure is particularly well suited for hosting training-only attention modules. This property is less directly available in YOLOv10 and YOLOv11 within their default configurations, owing to their fundamentally different architectural designs. Third, we validated the effectiveness of our proposed method using a real-world industrial PPE detection dataset and demonstrated performance improvements in mean Average Precision compared with the baseline YOLOv9 and the YOLOv7, YOLOv8, and YOLOv11 series.

The structure of this study is as follows. Section 2 describes existing object detection techniques, the core concepts of the YOLOv9 model architecture, and related work on attention mechanisms. Section 3 details the design principles and integration method of the proposed CBAMLinear module based on YOLOv9’s PGI architecture. Section 4 presents the experimental environment and settings using industrial field data, evaluation metrics, and performance analysis results. Section 5 provides additional discussion and interpretation of the research results. Section 6 presents the conclusions of this study and future research directions.

The contributions of this study, as stated in the Introduction, are as follows:

- zero-overhead attention paradigm: CBAMLinear introduces an attention integration strategy that, to the best of our knowledge, among the YOLO variants surveyed in this study, provides zero inference-time cost. This is verified by direct measurement, which shows identical parameters and FLOPs between YOLOv9 without CBAM and YOLOv9 with CBAMLinear at 640×640 input resolution. No prior CBAM with YOLO work has achieved this property: YOLO-BAM, SCM-YOLO, YOLOv5-CBAM variants, and YOLOv8-CGS all incur permanent inference overhead [11,12].
- CBAMLinear design within YOLOv9’s auxiliary branch: The proposed CBAMLinear module replaces CBLinear in the training-only auxiliary reversible branch, enabling attention-enhanced gradient information without altering the inference-time computation graph.
- Real-world industrial validation: The proposed method is validated on a PPE detection dataset, achieving incremental but consistent mAP improvements over the baseline YOLOv9 for the three larger model variants, and surpassing YOLOv7, YOLOv8, and YOLO11 series under identical training conditions.

## 2. Related Work

### 2.1. Object Detection

In the field of computer vision, object detection technology automatically identifies specific targets in images or videos and estimates their locations. It serves as a core element in various application areas such as autonomous driving, intelligent surveillance systems, and medical image analysis [13,14,15]. As application demands grow across these diverse fields, object detection models have evolved to achieve both accuracy and speed [16]. In early object detection, the sliding window technique was primarily used to scan the entire image at regular intervals. For each window, manual features like Histogram of Oriented Gradient (HOG) or Haar-like features were extracted, then classified by a classifier. While this approach showed reasonable performance in controlled environments, its performance differed significantly when applied to real-world scenarios. Factors like lighting changes and complex backgrounds caused false positives and false negatives to surge dramatically. Moreover, computational load increased proportionally with the number of windows, making practical implementation difficult [17].

However, these limitations changed with the advent of deep learning, particularly Convolutional Neural Networks (CNNs). CNNs hierarchically represent visual features in images, from low-level to high-level features [18,19]. This characteristic of CNNs enabled the recognition of objects with varying sizes and shapes. Subsequently, two-stage methods like the R-CNN series, which use CNNs on candidate regions, and one-stage methods like SSD and YOLO, which predict boxes and classes simultaneously using a single CNN, emerged. Object detection saw improvements in both accuracy and speed [4,20,21].

These architectures have also been extensively applied to personal protective equipment detection. One study proposed a domain-adaptive Faster R-CNN for non-PPE identification on construction sites, integrating image-level and instance-level adversarial domain classifiers to address the scarcity of genuine non-PPE instances [22]. Another work systematically compared Faster R-CNN, RetinaNet, and YOLOv5 within a two-step helmet-wearing detection framework, reporting that the choice of detection architecture significantly affects performance across varying scene conditions [23]. A separate study proposed an SSD-based safety helmet detection method with a cross-layer attention mechanism and multiscale perception module [24]. For anchor-free approaches, prior work introduced RBFPDet, which reformulates helmet detection as a semantic feature point detection task [25]. More recently, a multi-baseline study benchmarked Faster R-CNN, SSD, and RetinaNet against an improved YOLOv8 on the SHWD dataset, providing a comprehensive comparison across detector series [26]. The PPE detection approaches surveyed above can be organized along two axes: detection architecture and attention usage. Two-stage methods such as domain-adaptive Faster R-CNN offer high accuracy but are insufficiently fast for real-time industrial monitoring. One-stage detectors including SSD-based and YOLO-based approaches achieve real-time speed but sacrifice accuracy on small or occluded PPE objects. Recent attention-enhanced one-stage methods improve accuracy by embedding attention in the main inference path, but invariably increase computational cost. None of the existing approaches achieves both accuracy improvement through attention and zero-inference overhead, which is the gap addressed by the present work.

Transformer-based DETR demonstrated end-to-end learning without anchors or NMS through self-attention, pointing to a new direction [27,28].

YOLO became a representative model for real-time detection by dividing images into grids and simultaneously regressing the bounding box and class for each grid using a single neural network [4]. From YOLOv2 to YOLOv4, it employed an anchor-based structure that predefined anchor boxes of multiple scales and predicted classes for each anchor at its respective location [29,30]. While this improved accuracy, it introduced issues such as cumbersome hyperparameter tuning for anchor configurations and increased output dimensions and post-processing costs. To mitigate these problems of anchor-based methods, research on anchor-free approaches has recently flourished. Models like CornerNet, CenterNet, and FCOS directly predict object center points, corners, pixel-level locations, and box sizes instead of anchors. Furthermore, they eliminated dependence on anchor design, enabling the network to learn location information more flexibly from the data. In practice, they demonstrated outstanding performance in complex scenes and environments with objects of varying scales while also reducing design and implementation complexity.

YOLO has thus continued to evolve. YOLOv5 retained anchors, while YOLOv6 advanced to an anchor-free approach [31,32]. Furthermore, YOLOv7 redesigned the backbone and neck, further improving the balance between accuracy and speed through data augmentation and training stabilization techniques [33]. YOLOv8 simplified the output structure by adopting an anchor-free head as standard, reducing post-processing costs while maintaining strong performance across multiple datasets [5].

This study proposes a method for detecting personal protective equipment in industrial settings based on YOLOv9. YOLOv9 is a model that mitigates information loss and enhances computational efficiency through its PGI and GELAN structures. This research further integrates CBAM to improve detection performance in complex industrial environments.

### 2.2. YOLO

As deep learning-based object detection models advance, the depth of the networks also increases accordingly. As network depth increases, the problem of the information bottleneck phenomenon also intensifies [34]. This problem arises because key feature information extracted in the early layers is lost or distorted during transmission to deeper layers, adversely affecting learning.

To address these issues, YOLOv9 introduced the Programmable Gradient Information (PGI) structure and the Generalized Efficient Layer Aggregation Network (GELAN) structure [8]. The PGI structure introduced in the YOLOv9 model incorporates an auxiliary reversible branch to address information loss due to the bottleneck phenomenon. This branch preserves feature information extracted from early layers while reliably propagating gradients, thereby enhancing stability during training [35]. YOLOv9’s GELAN structure generalizes YOLOv7’s Efficient Layer Aggregation Network (ELAN), supporting and integrating various computational blocks [33]. It also employs the efficiency concept from Cross Stage Partial Network (CSPNet) to reduce redundant computations [36]. GELAN optimizes information flow within the model, significantly reducing computational load and parameter count to prevent performance degradation. It is designed to satisfy both computational efficiency and expressiveness.

In the YOLOv9 model, the PGI plays a crucial role in detecting objects of various scales. The PGI structure is designed to suppress feature information loss, which frequently occurs when detecting small objects, by inserting auxiliary information across multiple layers of the Feature Pyramid Network (FPN) [37]. Furthermore, PGI’s auxiliary branch reintroduces gradient information that might otherwise be lost in the main network, contributing to training stability. In this manner, the PGI structure enhances the overall training stability of the model and enables learning for objects of diverse sizes. Through PGI and GELAN, YOLOv9 mitigates the information loss issues plaguing previous models while demonstrating improved performance. YOLOv9 is designed to simultaneously achieve model lightweighting and high detection accuracy due to its PGI and GELAN structures [8]. Among recent YOLO variants, however, the architectures differ fundamentally in how they handle auxiliary information during training. YOLOv7 introduced E-ELAN for gradient path optimization but does not maintain a training-only auxiliary branch that is removed at inference. YOLOv8 adopted an anchor-free head and simplified the output structure but follows a single-branch design without a parallel auxiliary path. YOLOv10 introduced a consistent dual assignment strategy for NMS-free inference, which operates as a label assignment mechanism rather than a feature-processing branch. YOLOv11 integrates C2PSA attention directly into the main inference pipeline, permanently increasing computational cost. Among the YOLO variants surveyed in this study, YOLOv9’s PGI architecture is the one that maintains a training-only auxiliary reversible branch structurally separable from the inference path via reparameterization, which makes it structurally suited for hosting attention modules with zero deployment overhead.

### 2.3. Attention Mechanism

The attention mechanism is a structure that enables selective focus on specific regions or features within input data [38,39]. The attention mechanism first gained prominence in natural language processing. It was initially introduced to address information loss issues when processing long sentences in sequence-to-sequence models for translation [40,41]. Information loss frequently occurs in encoder-decoder architectures. To resolve this bottleneck, the attention mechanism enabled the decoder to access every position in the input sequence at each time step, allowing it to dynamically focus on contextually relevant information.

Subsequently, the attention mechanism became a core component of the Transformer architecture. The self-attention mechanism proposed in Transformer models improved learning efficiency by enabling parallel computation of global dependencies between all positions within a sequence, rather than the sequential processing approach used previously. This improvement went beyond mere computational speed enhancement; it enabled effective modeling of long-term dependencies, leading to outstanding performance even in learning complex patterns [39,42].

In the field of object detection, the Detection Transformer (DETR) model, a Transformer-based approach utilizing attention mechanisms, has emerged [27]. DETR reframes object detection as a set prediction problem, eliminating complex post-processing steps like anchor boxes and non-maximum suppression that were essential in previous methods [28]. While this approach simplifies the structure and is designed to learn global relationships and interactions between objects, it suffers from substantial computational cost and long training times [27].

Meanwhile, in the field of CNN-based object detection, various studies are underway to efficiently utilize attention mechanisms [43]. Representative examples include modules such as Squeeze-and-Excitation (SE) and the Convolutional Block Attention Module (CBAM). SE learns inter-channel dependencies to calibrate feature responses per channel, while CBAM sequentially applies channel and spatial attention to progressively refine intermediate feature maps [44,45,46].

In particular, according to the experimental results in existing CBAM papers, applying both channel attention and spatial attention sequentially demonstrated superior performance compared with using a single module or applying them in parallel [47]. Based on this validated design principle, this study adopts the CBAM module, designed to enable the model to selectively focus on informative features along both the channel “what” and spatial “where” dimensions in a omplementary manner.

CBAM has been extensively integrated with YOLO-series models across multiple generations. YOLO-BAM inserted CBAM into the YOLOv3 backbone for pedestrian detection [11]. SCM-YOLO embedded CBAM in the YOLOv4-tiny backbone for safety helmet detection, achieving a 4.36% mAP improvement with permanent inference overhead [48]. In the YOLOv5 series, adopting CBAM into the FasterNet architecture for object detection reported an additional 10.2% in parameters with no extra FLOPs, while measuring a 25% increase in inference-time [12]. Across all these works, a consistent pattern emerges: CBAM is universally placed in the main inference pipeline backbone, neck, or head and remains active during deployment, invariably adding computational overhead.

Table 1 summarizes representative attention-integrated YOLO variants along four axes, backbone version, attention module location, activeness at inference, and additional inference-time cost, to clarify how the proposed CBAMLinear differs structurally from prior works.

As summarized in Table 1 and shown in the results of the above studies, all existing methods that integrate CBAM with the YOLO series have, by placing attention mechanisms within the main inference pipeline, inherently tied accuracy improvements and computational overhead together in an inseparable relationship. In contrast, this study leverages the unique architectural characteristics of YOLOv9’s PGI. Specifically, the auxiliary reversible branch constitutes an independent feature processing path that exists only during training and is completely removed during the inference stage through reparameterization. The CBLinear modules within this branch receive features from the main backbone via 1×1 convolutional operations but perform only simple linear transformations without considering feature importance. By replacing CBLinear with CBAMLinear, we enrich the features sent to the auxiliary branch via channel and spatial attention, and generate attention-based gradients that improve the learned representations of the main branch during backpropagation. This design improves accuracy while maintaining the same inference architecture, parameters, and FLOPs as the original YOLOv9, a property that, to the best of our knowledge, has not been demonstrated by the CBAM-integrated YOLO variants surveyed.

Therefore, the attention integration strategies of the YOLO series models can be classified into two paradigms. The first paradigm, which encompasses all the prior studies listed in the table, embeds the attention module into the backbone, neck, or head of the main inference pipeline, keeping it active even during deployment and thereby permanently increasing the inference cost. The second paradigm proposed in this study inserts the attention mechanism only into a training-only auxiliary branch and removes it via re-parameterization during inference, thereby decoupling accuracy improvements from inference overhead. This classification of the two paradigms highlights that the novelty of this study lies not in the choice of attention modules but in the integration paradigm itself.

This study aims to leverage the proven effectiveness of this attention mechanism within the YOLOv9 model. The goal is to enhance object detection performance by introducing CBAM into the auxiliary reversible branch of the PGI architecture. Through CBAM’s channel and spatial attention functions, we expect to make the information transmitted to the auxiliary reversible branch more distinct and stabilize the gradient flow, ultimately enhancing the overall model’s learning quality. This approach is well suited to scenarios demanding both high accuracy and real-time detection, such as personal protective equipment detection in industrial settings.

## 3. YOLOv9-CBAMLinear Based Object Detection

### 3.1. Model Structure

**Figure 1:**
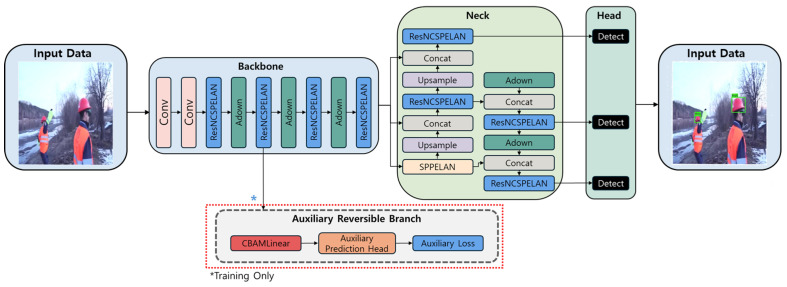
YOLOv9 architecture with the proposed CBAMLinear modification. The dotted boundary encloses the PGI auxiliary reversible branch, which is active only during training and is removed at inference via reparameterization. CBAMLinear replaces CBLinear at pyramid levels P3, P4, and P5 within this branch. All components outside the dotted boundary constitute the inference-time model, whose parameters and FLOPs are identical to the baseline YOLOv9. * denotes the PGI auxiliary reversible branch with the proposed CBAMLinear modification.

The structure of the proposed YOLOv9-CBAMLinear in this study is as shown in Figure 1. This study introduces a zero-inference overhead modification to YOLOv9. We replace only the CBLinear modules at levels P3, P4, and P5 within the auxiliary reversible branch with our CBAMLinear design. No other component of the YOLOv9 architecture backbone, neck, detection head, CBFuse modules, auxiliary backbone layers, or auxiliary detection heads is modified. Because this auxiliary branch is discarded during inference, the final model adds no computational burden to the baseline architecture. In the existing YOLOv9, features extracted from the backbone are passed to the auxiliary reversible branch of the PGI to reduce information loss in deep networks and stabilize gradient flow. However, the CBLinear module used in this process performs only a simple linear transformation and does not consider the varying importance of features across channels or spatial locations. This lack of consideration for feature importance limits the efficiency of information transfer through the auxiliary branch.

To address this limitation, this study designs a CBAMLinear module by combining CBAM with CBLinear and integrates it into PGI’s auxiliary branch. When features are transmitted through the auxiliary branch, CBAM emphasizes important features and suppresses unnecessary information. CBAMLinear is positioned within PGI’s auxiliary branch and selectively performs emphasis in both the channel and spatial dimensions during training. During inference, the auxiliary branch is deactivated, and CBAMLinear is also deactivated. This design improves learning quality while maintaining the same inference cost as the baseline YOLOv9.

In the overall pipeline of Figure 1, the backbone extracts multiscale features from the input image. The extracted features are split into two paths. One is the main branch, which goes directly to the detection head, and the other is the path through the PGI auxiliary branch. In the auxiliary branch, CBAMLinear refines the features and then provides gradient information back to the main branch. During training, the main branch receives high-confidence gradients from the auxiliary branch, enabling stable learning. At inference-time, the entire auxiliary branch is deactivated, leaving only the main branch. This branch connects directly to the detection head, producing results quickly. This means the number of parameters and computations at inference-time is identical to that of the original YOLOv9.

**Figure 2:**
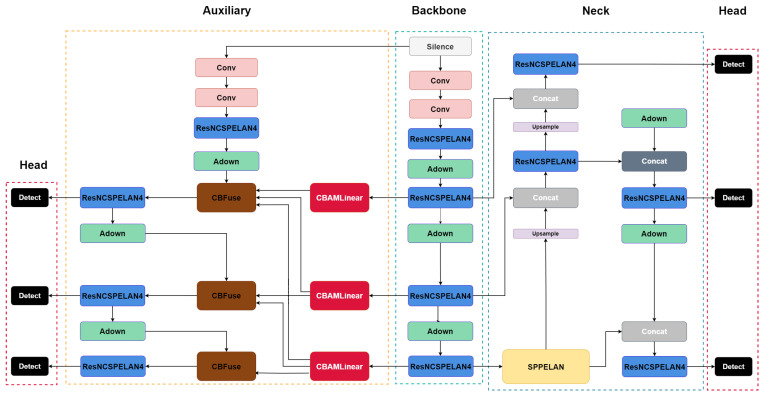
Module composition of the proposed YOLOv9-CBAMLinear model, showing the complete information flow during training where both the main path and auxiliary path are active, contrasted with the inference configuration in which only the main path remains. Backbone features are aggregated at multiple scales via upsampling and concatenation in the neck, and the auxiliary path applies CBAMLinear at each scale before being combined through CBFuse. The auxiliary path is removed via reparameterization at inference.

Figure 2 illustrates the complete information flow during training, where both the main path and the auxiliary path are active, contrasted with the inference configuration in which only the main path remains. Features extracted from the backbone are aggregated at multiple scales via upsampling and concatenation in the neck layer, and the final detection result is output through multiscale detection. The auxiliary path branches off from the backbone features, applies CBAMLinear at each scale, and combines the information using CBFuse to provide gradient signal during training. Because this entire auxiliary path is discarded via reparameterization at inference, the deployed model’s parameter count and FLOPs are identical to those of the baseline YOLOv9.

### 3.2. CBAMLinear Module

**Figure 3:**
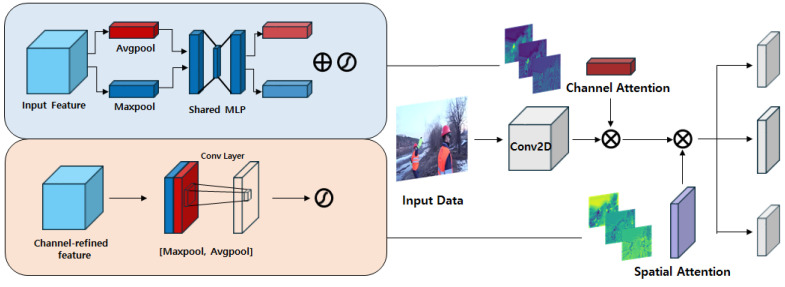
Detailed architecture of the proposed CBAMLinear module.

Figure 3 shows the structure of the CBAMLinear module proposed in this study. This module combines the existing CBLinear with CBAM, performing selective emphasis in the channel and spatial dimensions when passing features through the auxiliary reversible branch. CBAM is a module that emphasizes important features by sequentially applying channel attention and spatial attention. In this study, by integrating it with CBLinear, we improved the quality of features transmitted by the auxiliary branch using CBAM.

The original CBLinear module in YOLOv9’s PGI auxiliary branch performs a simple 1×1 linear projection that treats all channels and spatial locations uniformly, which does not exploit the fact that discriminative information for detecting small or low-contrast PPE objects is non-uniformly distributed across feature dimensions. To address this limitation, CBAMLinear inserts channel attention followed by spatial attention before the 1×1 projection, so that channels carrying strong discriminative signal and spatial regions corresponding to foreground PPE objects are selectively amplified. The sequential channel-then-spatial ordering follows the original CBAM design, which empirically demonstrated that this order outperforms parallel application or the reverse order. Because the resulting attention-refined features are then projected into the PGI auxiliary branch, whose loss is included with weight $\alpha_{aux}=0.25$ in the YOLOv9 dual loss computation, and because the backbone parameters are shared between the main and auxiliary branches, these attention-refined gradients contribute to the updates of the shared backbone parameters during backpropagation, despite the reduced auxiliary-branch weighting.

In the CBAMLinear module, the input feature map first passes through channel attention. At this stage, the importance of each channel is evaluated to compute the channel attention map. Channel-specific context is extracted via global average pooling and global max pooling, processed through a shared MLP, and then passed through a sigmoid to generate the channel attention map.

The CBAMLinear module processes an input feature map $F\in R^{C\times H\times W}$ as follows. First, channel attention is applied:

(1) $$M_{c}\left(F\right)=\sigma \left(\mathrm{MLP}\left(\mathrm{AvgPool}\left(F\right)\right)+\mathrm{MLP}\left(\mathrm{MaxPool}\left(F\right)\right)\right).$$

(2) $$F^{\prime }=M_{c}\left(F\right)⊗F.$$

where MLP denotes a shared two-layer fully connected network with a reduction ratio r=16, and σ is the sigmoid function. Next, spatial attention is applied:

(3) $$M_{s}\left(F^{\prime }\right)=\sigma \left({\mathrm{Conv}}_{7\times 7}\left(\left[{\mathrm{AvgPool}}_{c}\left(F^{\prime }\right);{\mathrm{MaxPool}}_{c}\left(F^{\prime }\right)\right]\right)\right).$$

(4) $$F^{\prime \prime }=M_{s}\left(F^{\prime }\right)⊗F^{\prime }.$$

where ${\mathrm{AvgPool}}_{c}$ and ${\mathrm{MaxPool}}_{c}$ denote pooling along the channel dimension, and ${\mathrm{Conv}}_{7\times 7}$ is a convolution with a 7×7 kernel. Finally, the attention-refined features are projected by the original CBLinear operation:

(5) $$\mathrm{Output}=\mathrm{Split}({\mathrm{Conv}}_{1\times 1}\left(F^{\prime \prime }\right)).$$

where ${\mathrm{Conv}}_{1\times 1}$ projects $F^{\prime \prime }$ to the required channel dimensions for the auxiliary branch, and Split divides the output along the channel axis to produce features matching each pyramid level of the auxiliary branch [47].

In summary, the CBAMLinear module produces a refined feature map in which object-relevant channels and spatial locations are selectively amplified before being projected into the auxiliary reversible branch. Unlike conventional attention integrations that embed such modules into the main inference path and thereby incur deployment-time overhead, the approach proposed in this study positions CBAMLinear exclusively within the PGI auxiliary branch, which is used solely for training. Thus, while CBAMLinear improves feature representation and gradient quality during training, it does not execute during inference because the entire auxiliary branch is deactivated. Consequently, it enhances training efficiency without increasing inference cost.

The role of CBAMLinear in the PGI architecture extends beyond simple feature highlighting. Since the purpose of the auxiliary branch is to compensate for information loss in the main branch’s deep network, it is more significant in selectively conveying important information that should be preserved through channel and spatial attention. Particularly in scenarios like detecting personal protective equipment in industrial settings, where small objects are embedded in complex backgrounds, CBAMLinear prioritizes emphasizing object-relevant channels and spatial regions, maximizing the auxiliary branch’s supportive effect. By concentrating gradient feedback on relevant features, the method proposed in this study significantly enhances the main branch’s ability to learn these small-object features. Furthermore, although the auxiliary branch loss is weighted by $\alpha_{aux}=0.25$ in the official YOLOv9 loss computation, the auxiliary branch still delivers a non-trivial gradient signal to the shared backbone parameters during backpropagation. Because of this structural role, the attention-refined gradients produced by CBAMLinear contribute to the updates of the shared backbone parameters even under the reduced auxiliary weighting. This structural prioritization ensures that the gradient signal flowing through the auxiliary branch becomes not only reliable, as in baseline PGI, but also selectively focused on the most informative channel and spatial dimensions. At the same time, the entire branch—including CBAMLinear—is eliminated at inference via reparameterization, yielding zero additional parameters, FLOPs, or latency.

### 3.3. Training and Inference Mechanism

This study adopts a method that replaces the CBLinear module within the auxiliary reversible branch of the PGI structure proposed in the existing YOLOv9 with CBAMLinear, which combines CBAM with the CBLinear module. CBAMLinear is designed to operate only within the PGI auxiliary reversible branch. Similar to YOLOv9’s design, it activates only during the training phase and deactivates during inference along with the entire auxiliary branch.

Our implementation follows the official YOLOv9 dual-branch training pipeline. The total loss combines three components for each branch: box regression, classification, and distribution focal loss following YOLOv9’s anchor-free design that eliminates the conventional objectness and confidence loss. Box regression loss is the Complete IoU (CIoU) loss:

(6) $$L_{box}=1-\mathrm{CIoU}\left(\hat{b},b\right).$$

where $\hat{b}$ and *b* denote the predicted and ground-truth bounding boxes.

Classification loss is binary cross-entropy with logits, applied to soft target scores generated by the Task-Aligned Assigner as implemented in the official YOLOv9 codebase, with parameters topk=10, α=0.5, β=6.0:

(7) $$L_{cls}=\mathrm{BCEWithLogits}\left(\hat{p},t_{\mathrm{TAL}}\right).$$

where $t_{\mathrm{TAL}}$ denotes the alignment-weighted soft targets.

Distribution Focal Loss (DFL) models bounding box distances as distributions over discretized bins and applies weighted cross-entropy on the two nearest integer bins of the target:

(8) $$L_{dfl}=-\left(\left(y_{i+1}-y\right)\log p_{i}+\left(y-y_{i}\right)\log p_{i+1}\right).$$

The single-branch loss combines these components with fixed weights:

(9) $$L_{branch}=\lambda_{box}L_{box}+\lambda_{cls}L_{cls}+\lambda_{dfl}L_{dfl}.$$

where $\lambda_{box}=7.5$, $\lambda_{cls}=0.5$, and $\lambda_{dfl}=1.5$, following the YOLOv9 default configuration. CBAMLinear is designed to operate only within the PGI auxiliary reversible branch. Similar to YOLOv9’s design, it activates only during the training phase and deactivates during inference along with the entire auxiliary branch [8].

During training, features extracted by the backbone branch are split into the main branch and the PGI auxiliary branch. The main branch computes a detection loss $L_{main}$ through the detection head, while the auxiliary branch computes $L_{aux}$ using features refined in the channel and spatial dimensions via CBAMLinear. Both branches employ independent Task-Aligned Assigners. The total training loss is:

(10) $$L_{total}=L_{main}+\alpha_{aux}L_{aux}.$$

where $\alpha_{aux}=0.25$. The gradients generated from $L_{aux}$, enriched by CBAMLinear’s channel and spatial attention, are backpropagated to the main branch through the PGI structure. This helps the model learn clearer representations for objects that are small or have low contrast against the background, such as personal protective equipment.

Furthermore, during inference, the auxiliary reversible branch and CBAMLinear are fully deactivated, so $L_{aux}$ is entirely absent and the number of parameters and computational load in the actual inference path remain identical to the baseline YOLOv9. Therefore, the proposed CBAMLinear module possesses the structural advantage of providing attention-based learning assistance only during training while enhancing accuracy during inference without incurring additional computational overhead [8].

## 4. Experiment and Results

### 4.1. Dataset and Implementation Details

This study conducted experiments on personal protective equipment detection using a YOLOv9 model integrated with the proposed CBAMLinear module. The 2984 images used in this study were sourced from the AI-Hub collection, a Korean government-operated public repository. The dataset spans distinct construction sites across several facility categories distributed across multiple geographic regions of South Korea. Within each site, images were captured from a variety of camera angles, elevations, and viewing distances, and under varied lighting and background conditions representative of real industrial operation. The dataset is annotated with three personal protective equipment classes: safety helmets, safety shoes, and safety gloves, which were selected because they represent the most commonly required PPE items in Korean industrial safety regulations. Each image is annotated with bounding boxes in YOLO format, with the annotations prepared using the labelImg tool (version 1.8.6, open-source: https://github.com/HumanSignal/labelImg, accessed on 1 May 2026). The source images exhibit a wide variety of environmental conditions, including different lighting levels, camera angles, worker postures, and background complexities, reflecting the variability present in real industrial deployment scenarios.

The dataset was split into training, validation, and test sets with a fixed 8:1:1 ratio, corresponding to approximately 2387 training images, 298 validation images, and 299 test images. The split was performed with a fixed random seed to ensure reproducibility. All three PPE classes are represented in all three subsets. During both training and inference, all images are resized to 640×640 pixels, following the standard YOLOv9 input configuration. While the dataset of 2984 images is smaller than general-purpose benchmarks such as MS COCO, its scale is consistent with domain-specific PPE detection studies, where annotated real-world industrial data is inherently costly to acquire due to restricted site access and safety-compliant data collection protocols. The images were captured across diverse real factory and construction site conditions rather than in controlled settings, and the strong augmentation pipeline described above partially compensates for the limited dataset size by generating composite training samples with increased visual diversity. Nevertheless, we acknowledge that the limited dataset scale constrains the strength of generalization claims that can be drawn from the reported results, and the conclusions of this study should accordingly be interpreted as evidence on a representative Korean industrial PPE dataset rather than as a guarantee of cross-domain robustness.

The training environment was configured with an AMD Ryzen 5 8645HS CPU (AMD, Santa Clara, CA, USA) and an NVIDIA GeForce RTX 3050 Laptop GPU (NVIDIA, Santa Clara, CA, USA), running on Windows 11 with CUDA v12.7, cuDNN 9.1.0, and Python 3.12.3. Training parameters included a batch size of 8, a total of 100 epochs, and an input image resolution of 640 × 640. Following the standard YOLO training protocol, we applied mosaic augmentation throughout the entire training process, deactivating it only during the final 15 epochs using close mosaic. This schedule allows the model to learn from composite multi-image training samples during the main training phase while adapting to naturally composed images prior to final evaluation. For the CBAMLinear module, the CBAM components were configured as follows: the shared MLP in channel attention uses a reduction ratio of r=16, and the spatial attention uses a 7×7 convolution kernel, following the original CBAM design [47]. All CBAM-specific parameters (shared MLP weights and spatial convolution kernel) were randomly initialized using Kaiming initialization and trained from scratch alongside the entire model. No pretrained weights were loaded for any model, including the baseline YOLOv9, to ensure a fair comparison under identical initialization and training conditions.

All hyperparameters listed in Table 2 follow the official YOLOv9 high-augmentation configuration. This was a deliberate methodological choice: the contribution of this study is the architectural modification introduced by CBAMLinear within the PGI auxiliary branch, not a new hyperparameter tuning strategy. Retaining the official configuration eliminates the risk that the reported improvements stem from dataset-specific hyperparameter optimization rather than from the proposed architectural contribution, and ensures direct comparability with the published YOLOv9 results reported in the original YOLOv9 paper. All comparison models were similarly trained using their respective official hyperparameter configurations, with common factors batch size, epochs, input resolution, and data augmentation unified across all models to ensure a fair comparison.

The complete data augmentation pipeline follows the official YOLOv9 high-augmentation configuration, including Mosaic augmentation (probability 1.0, disabled in the final 15 epochs via close-mosaic), MixUp (probability 0.15), Copy-Paste augmentation, HSV color space jitter, random horizontal flip, and random translation and scaling. These augmentation parameters are held identical across all comparison models to ensure fair evaluation conditions.

The complete dataset preparation scripts, split configuration files, and training code are available at https://github.com/OOhoy/YOLOv9-CBAM (accessed on 1 May 2026) for review. Furthermore, the dataset was split into train/validation/test sets at an 8:1:1 ratio for model evaluation. Evaluation metrics included mAP@0.5 and mAP@0.5:0.95.

### 4.2. Evaluation Metrics

The evaluation metrics for this study are mAP@0.5 and mAP@0.5:0.95, which are standard for object detection benchmarks. Average Precision (AP) is computed as the area under the Precision–Recall curve, and mean Average Precision (mAP) is obtained by averaging AP over all classes. Following the COCO benchmark convention:

- mAP@0.5: mean Average Precision at an IoU threshold of 0.5
- mAP@0.5:0.95: mean Average Precision averaged over IoU thresholds from 0.5 to 0.95 in steps of 0.05.

### 4.3. Experimental Setup

The experiments in this study aimed to verify the detection performance of personal protective equipment for worker safety in industrial environments, evaluating the effectiveness of a structure integrating CBAM into YOLOv9. The experiments were conducted in the following three aspects.

First, we compared the baseline model YOLOv9 with the YOLOv9 model combined with CBAMLinear to analyze the quantitative performance improvement achieved by integrating CBAM. This measured the direct contribution of the proposed module. Second, the relative performance advantage of combining YOLOv9 with CBAM was confirmed by comparing it with the YOLOv7 model, which shares a similar architecture to the baseline YOLOv9 model. Third, the competitiveness of the proposed method was evaluated by comparing it with the state-of-the-art model, YOLOv11. YOLOv11, a validated and recently proposed model in the YOLO series, was included to verify the performance level achieved by this study’s approach relative to state-of-the-art techniques. All comparison models were trained on the same dataset, under identical training conditions and preprocessing methods to ensure a fair comparison. Evaluation metrics included mAP@0.5 and mAP@0.5:0.95.

### 4.4. Results and Analysis

Using the evaluation metrics defined in Section 4, the detection performance of the proposed YOLOv9-CBAMLinear model was comprehensively evaluated. Experiments were conducted in three aspects: analyzing the effectiveness of CBAMLinear, comparing with architecturally similar models, and comparing with the latest models.

In the results tables throughout this section, the suffixes attached to the YOLOv9 model names denote the size variants of the YOLOv9 series released in the official implementation. Specifically, YOLOv9-t, YOLOv9-s, YOLOv9-m, YOLOv9-c, and YOLOv9-e correspond to the tiny, small, medium, compact, and extended variants, respectively, which differ in the depth and width scaling of the GELAN backbone and the PGI auxiliary branch.

Before analyzing detection performance, we first verify the computational cost of the proposed method. Table 3 presents the parameter counts and FLOPs for all comparison models, measured at 640 × 640 input resolution. A critical observation is that the inference-time, parameter and FLOPs of YOLOv9-CBAMLinear models are identical to those of the corresponding baseline YOLOv9 models across all variants. This confirms that CBAMLinear introduces zero computational overhead during deployment, as the entire auxiliary reversible branch containing CBAMLinear is removed via reparameterization. This result validates the practical viability of the proposed method for real-time deployment in resource-constrained industrial environments.

First, comparing cases with and without the Convolutional Block Attention Module applied to the YOLOv9 model series showed improvements in mAP@0.5 and mAP@0.5:0.95 for the three larger variants. The YOLOv9-c and YOLOv9-m variants similarly showed consistent improvements under CBAMLinear integration, with mAP@0.5:0.95 increase.

The critical observation in Table 3 is that the #Param. (M) FLOPs and FPS columns are identical between the without CBAM and with CBAM rows for every YOLOv9 variant, confirming zero-inference overhead for the proposed method. In terms of accuracy, CBAMLinear integration improves mAP@0.5 and mAP@0.5:0.95 for the three larger variants (e, c, m). Since the proposed YOLOv9-CBAMLinear shares an identical inference-time computation graph with the baseline YOLOv9 after reparameterization, the inference-time efficiency of the baseline YOLOv9 transfers directly to the proposed method on any hardware. We measured the inference frame rate (FPS) of all five YOLOv9 variants, with and without CBAMLinear, on the evaluation hardware described in Section 4.1 at 640 × 640 input resolution; the results are reported in the rightmost column of Table 3. Across all variants, the baseline YOLOv9 and YOLOv9-CBAMLinear achieve identical FPS, providing direct empirical confirmation that CBAMLinear introduces no inference-time overhead after reparameterization. The heaviest variant, YOLOv9-e, sustains 38 FPS, while YOLOv9-c, m, s, and t reach 56, 62, 72, and 76 FPS, respectively.

YOLOv7 shares similar structural characteristics with YOLOv9, prompting a performance comparison. Table 4 compares the performance of YOLOv9 variants with CBAMLinear against models from the YOLOv7 series. All five YOLOv9 variants with CBAMLinear achieved mAP@0.5:0.95 values above the 0.302–0.532 range observed across the six YOLOv7 variants, with YOLOv9-e reaching 0.658 ± 0.003. The gap between YOLOv9-e (0.658) and the best YOLOv7 variant corresponds to approximately +12.6 percentage points, indicating that the combination of YOLOv9’s PGI architecture and the proposed CBAMLinear module exceeds the mAP@0.5:0.95 performance of the YOLOv7 family on this PPE dataset under identical training conditions.

As a representative one-stage detector, YOLOv8 serves as a robust baseline for validating the proposed model. Table 5 presents the results comparing the quantitative performance of the proposed model, which integrates CBAMLinear into YOLOv9, against YOLOv8. The three larger YOLOv9 variants with CBAMLinear achieve mAP@0.5:0.95 values of 0.658, 0.649, and 0.645, respectively, substantially higher than the 0.575–0.582 range observed across YOLOv8 variants.

The comparison in Table 5 is particularly important because YOLOv8 was released shortly before YOLOv9 and represents a directly competing architectural design. The three YOLOv9 variants with CBAMLinear achieved mAP@0.5:0.95 values of 0.658, 0.649, and 0.645, respectively, above the 0.575–0.582 range observed across all five YOLOv8 variants. This indicates that the advantage reflects the specific contribution of CBAMLinear within YOLOv9’s PGI structure rather than being merely a matter of backbone generation.

YOLOv11 is a recently released model in the YOLO series, with five variants covering lightweight to large configurations. According to the performance comparison results presented in Table 6, the YOLOv9-e with CBAMLinear proposed in this study achieved mAP@0.5:0.95 of 0.658 ± 0.003, higher than all YOLOv11 variants, the highest of which is YOLOv11-m at 0.629 ± 0.001. This indicates that the attention mechanism integrated into YOLOv9’s PGI auxiliary branch enables effective feature learning comparable to or exceeding what the YOLOv11 architecture achieves on this PPE dataset. The YOLOv9-e with CBAMLinear achieved the highest mAP@0.5 (0.964) and mAP@0.5:0.95 (0.658) among all compared variants, while adding zero inference-time computational cost.

This finding is particularly significant because YOLOv11 already integrates an attention mechanism into its main inference path and therefore bears additional inference-time cost. The proposed CBAMLinear approach, which adds zero inference cost, still outperforms YOLOv11 on this PPE dataset. This demonstrates that training-time-only attention enrichment within the PGI auxiliary branch can be more effective than continuously active attention in the main inference path, at least for the scenarios considered in this study.

### 4.5. Per-Class Detection Performance

To directly evaluate how well each of the three target PPE classes is detected, Table 7 reports per-class mAP@0.5 and mAP@0.5:0.95 for YOLOv9 with CBAMLinear across all five model scales.

Three observations follow from Table 7. First, the three PPE classes exhibit a consistent difficulty ordering across all five model scales: Safety Helmet achieves the highest mAP, followed by Safety Gloves, and then Safety Shoes. This ordering reflects the progressively smaller apparent size and increasing frequency of occlusion from helmets to gloves to shoes. Second, Safety Helmet detection is effectively saturated on this dataset, with mAP@0.5 exceeding 0.98 across all scales; remaining improvements in aggregate mAP therefore come primarily from the gloves and shoes classes. Third, this class-wise difficulty pattern is consistent with the per-class miss-as-background rates of 7%, 36%, and 57% for helmets, gloves, and shoes, respectively, as further detailed in the confusion matrix analysis presented later in this section.

**Figure 4:**
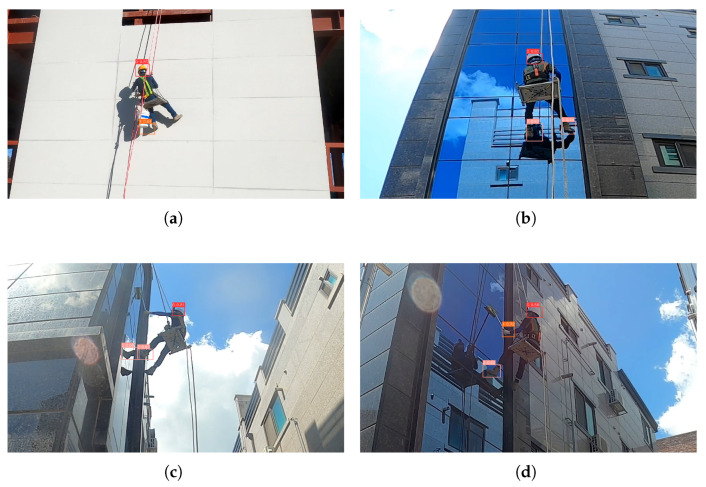
Qualitative detection results of YOLOv9 with CBAMLinear. (**a**,**c**) failure cases showing two distinct false-positive modes: shape ambiguity and specular reflection; (**b**,**d**) successful detections across multiple PPE classes. (**a**) False positive: bucket with cast shadow misdetected as gloves. (**b**) Success: helmet and shoes correctly detected. (**c**) False positive: glass facade reflection misdetected as shoes. (**d**) Success: helmet, gloves, and shoes all correctly detected.

Figure 4 presents four representative qualitative detection cases covering the three target PPE classes. The two successful cases show the proposed YOLOv9 with CBAMLinear correctly detecting safety helmets, safety gloves, and safety shoes across multiple industrial site conditions. The two challenging cases illustrate two distinct false positive failure modes: a bucket with its cast shadow misdetected as safety gloves, and reflections of the worker’s feet on a glass facade misdetected as additional safety shoes. These qualitative examples indicate that remaining failure modes include shape-ambiguity and specular reflection conditions under which non-PPE objects can be confused with real PPE items.

**Figure 5:**
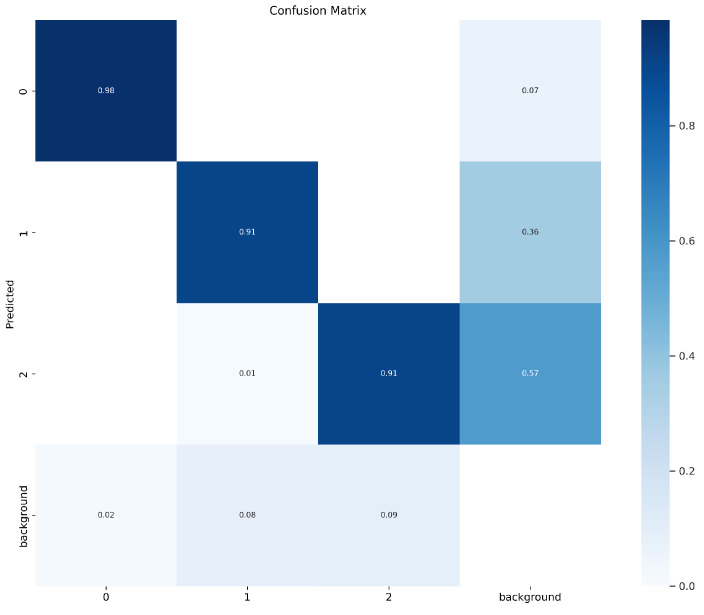
Confusion matrix of YOLOv9 with CBAMLinear.

Figure 5 presents the confusion matrix of YOLOv9 with CBAMLinear on the PPE test set. Safety helmets achieve the highest correct-detection rate, with only 7% missed as background. Safety gloves and safety shoes both achieve correct detection rates of 0.91 but show substantially higher miss-as-background rates of 36% and 57%, respectively. Cross-class misclassifications between the three PPE classes remain below 2%, indicating that remaining errors are dominated by missed detections and by false positives arising from visual-cue ambiguities rather than by confusion between PPE classes themselves.

The consistent improvement in the mAP@0.5:0.95 observed in CBAM using YOLOv9 aligns with the view that interprets CBAMLinear as a gradient-boosting mechanism rather than a feature extractor at the inference stage. Because CBAMLinear is removed from the inference graph via reparameterization, its only contribution to the trained model is the channel and spatial attention-refined gradient that flows through the PGI auxiliary branch into the shared backbone parameters during training. The benefit of such gradient-enrichment may be more readily observable when the main branch backbone has sufficient representational capacity to encode the refined feature distinctions induced by these gradients. For the three larger variants, the observed gains are consistent with this interpretation. For the two smallest variants, the differences across configurations fall within the run-to-run variance reported in Table 3, so we do not claim improvement at these scales. This scale-dependent pattern is also visible in the ablation results in Table 8, where the joint channel and spatial configuration tends to yield the highest mAP@0.5:0.95 among the four ablation settings for the three larger variants.

From a practical standpoint, the observed mAP@0.5:0.95 gains of 0.005–0.007 are modest in absolute terms but may carry additional value in the industrial safety context. In PPE monitoring, false negatives—that is, missed detections of unworn safety helmets, gloves, or shoes correspond to undetected safety violations, and reductions in the rate of such missed detections may contribute to safer operation when accumulated across continuous monitoring deployments, though the size of this effect would need to be evaluated in a deployed setting and is not measured in the present study. The per-class results in Table 7 and the confusion matrix analysis in Figure 5 indicate that gloves and shoes retain substantially higher miss-as-background rates than helmets, and accuracy gains on these classes are therefore a particularly relevant component of the reported improvement. Furthermore, because CBAMLinear introduces no additional inference-time parameters or FLOPs relative to the baseline YOLOv9, these accuracy gains can be obtained without changes to the computational profile of existing CCTV-based monitoring pipelines, which may lower the integration barrier for deployment in safety management systems operating under fixed hardware budgets.

### 4.6. Ablation Study

To isolate the respective contributions of channel attention and spatial attention within CBAMLinear, we conducted ablation experiments on the YOLOv9 base model under identical training conditions—same dataset split, same augmentation, same optimizer, and same learning rate schedule as described in Section 4.1. All ablation variants share the same inference architecture as the baseline YOLOv9, since all attention modules are inserted exclusively in the PGI auxiliary reversible branch.

As shown in Table 8, CBAM configuration of CBAMLinear improves mAP@0.5 and mAP@0.5:0.95 over the baseline YOLOv9 for the three larger variants (e, c, m), with mAP@0.5:0.95 gains of +0.007 for YOLOv9-e, +0.007 for YOLOv9-c, and +0.010 for YOLOv9-m. Channel attention alone and spatial attention alone also produce improvements over the baseline for these three variants, but the joint configuration most reliably yields the highest mAP@0.5:0.95, indicating that the channel and spatial components contribute complementary signals to the auxiliary branch’s gradient-enrichment. For the two smallest variants (YOLOv9-s, YOLOv9-t), differences among the four configurations fall within the standard deviation observed over three independent runs, suggesting that at small model capacity, the auxiliary branch provides only marginal additional benefit from attention-based refinement. This scale-dependent pattern is consistent with the interpretation that CBAMLinear operates as a gradient-enrichment mechanism whose effect is most visible when the main branch has sufficient capacity to absorb the refined gradient signal.

## 5. Discussion

The accuracy improvement reported in this study should be positioned as an incremental enhancement rather than a large performance breakthrough. The observed mAP@0.5:0.95 gain of 0.005–0.007 over the baseline YOLOv9 is modest in absolute terms, and the principal methodological value of CBAMLinear lies in coupling this incremental gain with zero inference-time overhead. With this scope in mind, this study has the following limitations and future directions. First, the dataset used in this study comprises 2984 images sourced from a multi-site AI-Hub collection spanning multiple facility categories and geographic regions. While this scale is consistent with comparable domain-specific PPE detection studies, it is small in absolute terms relative to general-purpose detection benchmarks, and this places an inherent limit on the generalization claims that can be drawn from the reported results. Although this multi-site composition provides within-dataset environmental diversity, no formal cross-dataset validation using additional public PPE benchmarks such as SHWD, SH17, or SFCHD was performed [49,50]. In future work, we plan to conduct cross-dataset validation on these benchmarks, providing a direct assessment of the proposed method’s external validity and domain generalization capability. Regarding lighting and occlusion robustness, although the AI-Hub source dataset provides within-distribution diversity across indoor factory floors, outdoor construction sites, and mixed-lighting scenarios, a formal evaluation under controlled lighting conditions and a systematic robustness analysis stratified by occlusion level were not conducted. These controlled robustness analyses are acknowledged as limitations to be addressed in future work.

Second, the present study focuses on the CBAMLinear design within the PGI auxiliary branch and does not include a systematic comparison against alternative attention modules within the same auxiliary-branch insertion paradigm, comparisons with non-YOLO detection architectures such as DETR and RT-DETR, or a sensitivity analysis of key hyperparameters [45,51,52]. These methodological extensions would further characterize which attention series are most effective as gradient-enrichment mechanisms, how the proposed paradigm compares to fundamentally different detection approaches, and how robust CBAMLinear is under off-default training regimes.

Third, although the zero-overhead property of CBAMLinear has been verified by direct measurement on the evaluation hardware, no deployment test in a real-world CCTV-based industrial monitoring environment has been conducted, and detailed error analysis such as failure case examination and confusion matrix analysis has not been performed. We plan to evaluate the proposed method on real CCTV footage from active industrial sites and to characterize deployment performance across additional hardware platforms, alongside a systematic error analysis to identify remaining detection failure modes.

The confusion matrix analysis presented in Figure 5 highlights that small-scale PPE classes remain a persistent challenge even after the CBAMLinear refinement, with miss-as-background rates for safety gloves and safety shoes substantially higher than those for safety helmets. This pattern indicates that the scale-dependent improvements observed in Table 3, while consistent across the three larger YOLOv9 variants, do not translate uniformly across object classes of differing apparent size. We therefore report this class-wise limitation transparently and leave targeted small-object enhancement—for example, multi-scale feature fusion tailored to gloves and shoes, or auxiliary branch variants that emphasize high-resolution spatial attention—as a direction for subsequent work.

## 6. Conclusions

This research proposes a personal protective equipment detection technique by combining CBAM with the YOLOv9 model as a solution to ensure worker safety in industrial settings. The proposed CBAMLinear module replaces the CBLinear modules at pyramid levels P3, P4, and P5 within YOLOv9’s PGI auxiliary reversible branch, enriching gradient information during training while being completely removed at inference via reparameterization.

The experimental evaluation yielded four principal findings, each directly traceable to a specific table or figure in the manuscript body. First, the proposed CBAMLinear was verified by direct measurement to introduce zero additional parameters and zero additional FLOPs, which follows as a structural consequence of placing the module exclusively within the PGI auxiliary reversible branch that is removed during inference via reparameterization. Second, the integration of CBAMLinear yielded incremental but consistent improvements in mAP@0.5:0.95 over the baseline YOLOv9 for the three larger variants: YOLOv9-e from 0.652 to 0.658, YOLOv9-c from 0.644 to 0.649, and YOLOv9-m from 0.640 to 0.645. For the two smallest variants (YOLOv9-s and YOLOv9-t), we do not claim any improvement. Although the absolute magnitude of the gains is modest, they are obtained at zero deployment-time computational cost, which we regard as the principal practical contribution of this work. Third, the ablation study confirmed that channel and spatial attention each contribute improvements over the baseline, with the joint configuration most reliably yielding the highest mAP@0.5:0.95 for the three larger variants. Fourth, when compared against YOLOv7, YOLOv8, and YOLOv11 series under identical training conditions on the same PPE dataset, YOLOv9 with CBAMLinear achieved the highest mAP across all compared variants, surpassing the best YOLOv11 variant despite YOLOv11 integrating its own attention mechanism in the main inference path.

In future research, we plan to conduct cross-dataset validation on public PPE detection benchmarks, including SHWD and SH17, to directly evaluate the external validity and domain generalization capabilities of the proposed method. We expect these extensions to further advance industrial safety management technology and contribute to ensuring worker safety.

## Figures and Tables

**Table 1:** Comparison of attention integrated YOLO studies with the proposed method.

Method	YOLO Version	Attention Location	Active at Inference	Extra Params/FLOPs
YOLO-BAM	YOLOv3	Backbone	✔	✔
SCM-YOLO	YOLOv4-tiny	Backbone	✔	✔
YOLOv5-CBAM	YOLOv5	Backbone/Neck	✔	✔
YOLOv8-CGS	YOLOv8	Main path	✔	✔
Ours (CBAMLinear)	YOLOv9	Aux. branch	× (training-only)	×

**Table 2:** Training hyperparameters used for all models in this study, following the official YOLOv9 high-augmentation configuration.

Hyperparameter	Value
Optimizer	SGD
Initial learning rate (${\mathrm{lr}}_{0}$)	0.01
Final learning rate factor (${\mathrm{lr}}_{f}$)	0.01
Learning rate schedule	Linear decay
Momentum	0.937
Weight decay	$5\times 10^{-4}$
Warmup epochs	3.0
Warmup momentum	0.8
Warmup bias LR	0.1
Batch size	8
Nominal batch size (accumulation target)	64
Total epochs	100
Close-mosaic epochs	15
Input resolution	640×640

**Table 3:** Comparison of YOLOv9 and YOLOv9-CBAMLinear.

Model	CBAMLinear	#Param. (M)	FLOPs (G)	FPS	mAP@0.5	mAP@0.5:0.95
YOLOv9-e	-	57.6	188.7	38	0.957 ± 0.001	0.652 ± 0.003
YOLOv9-e	✔	57.6	188.7	38	0.964 ± 0.004	0.658 ± 0.003
YOLOv9-c	-	25.2	101.8	56	0.954 ± 0.004	0.644 ± 0.002
YOLOv9-c	✔	25.2	101.8	56	0.961 ± 0.002	0.649 ± 0.001
YOLOv9-m	-	19.9	76.0	62	0.955 ± 0.002	0.640 ± 0.004
YOLOv9-m	✔	19.9	76.0	62	0.962 ± 0.002	0.645 ± 0.003
YOLOv9-s	-	7.0	26.2	72	0.937 ± 0.001	0.614 ± 0.001
YOLOv9-s	✔	7.0	26.2	72	0.936 ± 0.001	0.613 ± 0.001
YOLOv9-t	-	1.8	7.0	76	0.923 ± 0.001	0.574 ± 0.001
YOLOv9-t	✔	1.8	7.0	76	0.922 ± 0.001	0.573 ± 0.001

**Table 4:** Comparison of YOLOv9-CBAMLinear and the YOLOv7 series under identical training conditions on the PPE dataset at 640×640 input resolution. Reported values are mean ± std over three independent runs.

Model	CBAMLinear	mAP@0.5	mAP@0.5:0.95
YOLOv9-e	✔	0.964 ± 0.004	0.658 ± 0.003
YOLOv9-c	✔	0.961 ± 0.002	0.649 ± 0.001
YOLOv9-m	✔	0.962 ± 0.002	0.645 ± 0.003
YOLOv9-s	✔	0.936 ± 0.001	0.613 ± 0.001
YOLOv9-t	✔	0.922 ± 0.001	0.573 ± 0.001
YOLOv7	-	0.869 ± 0.001	0.500 ± 0.001
YOLOv7-x	-	0.897 ± 0.002	0.532 ± 0.001
YOLOv7-w6	-	0.724 ± 0.001	0.401 ± 0.001
YOLOv7-e6	-	0.775 ± 0.001	0.426 ± 0.001
YOLOv7-e6e	-	0.513 ± 0.002	0.302 ± 0.002
YOLOv7-tiny	-	0.890 ± 0.001	0.517 ± 0.001

**Table 5:** Comparison of YOLOv9-CBAMLinear and the YOLOv8 series under identical training conditions on the PPE dataset at 640×640 input resolution. Reported values are mean ± std over three independent runs.

Model	CBAMLinear	mAP@0.5	mAP@0.5:0.95
YOLOv9-e	✔	0.964 ± 0.004	0.658 ± 0.003
YOLOv9-c	✔	0.961 ± 0.002	0.649 ± 0.001
YOLOv9-m	✔	0.962 ± 0.002	0.645 ± 0.003
YOLOv9-s	✔	0.936 ± 0.001	0.613 ± 0.001
YOLOv9-t	✔	0.922 ± 0.001	0.573 ± 0.001
YOLOv8-n	-	0.939 ± 0.002	0.575 ± 0.002
YOLOv8-s	-	0.942 ± 0.002	0.582 ± 0.001
YOLOv8-m	-	0.938 ± 0.001	0.582 ± 0.001
YOLOv8-l	-	0.939 ± 0.002	0.582 ± 0.001
YOLOv8-x	-	0.944 ± 0.001	0.575 ± 0.002

**Table 6:** Comparison of YOLOv9-CBAMLinear and the YOLOv11 series under identical training conditions on the PPE dataset at 640×640 input resolution. Reported values are mean ± std over three independent runs.

Model	CBAMLinear	mAP@0.5	mAP@0.5:0.95
YOLOv9-e	✔	0.964 ± 0.004	0.658 ± 0.003
YOLOv9-c	✔	0.961 ± 0.002	0.649 ± 0.001
YOLOv9-m	✔	0.962 ± 0.002	0.645 ± 0.003
YOLOv9-s	✔	0.936 ± 0.001	0.613 ± 0.001
YOLOv9-t	✔	0.922 ± 0.001	0.573 ± 0.001
YOLOv11-n	-	0.932 ± 0.002	0.590 ± 0.003
YOLOv11-s	-	0.961 ± 0.001	0.615 ± 0.002
YOLOv11-m	-	0.963 ± 0.001	0.629 ± 0.001
YOLOv11-l	-	0.962 ± 0.001	0.623 ± 0.001
YOLOv11-x	-	0.960 ± 0.001	0.626 ± 0.002

**Table 7:** Per-class detection performance of YOLOv9 with CBAMLinear.

Scale	Helmet		Gloves		Shoes	
	mAP@0.5	mAP@0.5:0.95	mAP@0.5	mAP@0.5:0.95	mAP@0.5	mAP@0.5:0.95
YOLOv9-t	0.985	0.646	0.902	0.608	0.839	0.460
YOLOv9-s	0.986	0.685	0.938	0.652	0.891	0.508
YOLOv9-m	0.991	0.709	0.963	0.682	0.936	0.547
YOLOv9-c	0.988	0.708	0.959	0.685	0.924	0.550
YOLOv9-e	0.987	0.710	0.960	0.684	0.925	0.546

**Table 8:** Ablation study of channel and spatial attention components in CBAMLinear.

Model	Channel Attention	Spatial Attention	mAP@0.5	mAP@0.5:0.95
YOLOv9-e	-	-	0.957	0.650
	✔	-	0.956	0.641
	-	✔	0.957	0.649
	✔	✔	0.964	0.657
YOLOv9-c	-	-	0.952	0.643
	✔	-	0.960	0.645
	-	✔	0.956	0.643
	✔	✔	0.962	0.650
YOLOv9-m	-	-	0.954	0.637
	✔	-	0.960	0.644
	-	✔	0.955	0.643
	✔	✔	0.963	0.647
YOLOv9-s	-	-	0.937	0.614
	✔	-	0.936	0.612
	-	✔	0.937	0.616
	✔	✔	0.936	0.614
YOLOv9-t	-	-	0.923	0.574
	✔	-	0.922	0.573
	-	✔	0.923	0.574
	✔	✔	0.922	0.573

## Data Availability

This research used datasets from ‘Real-time video data from aerial work sites (AI-Hub, S. Korea)’. All data information can be accessed through ‘AI-Hub (https://www.aihub.or.kr, accessed on 1 May 2026)’.
